# Establishing a single-sex controlled human *Schistosoma mansoni* infection model for Uganda: protocol for safety and dose-finding trial

**DOI:** 10.1093/immadv/ltad010

**Published:** 2023-07-20

**Authors:** Andrew Abaasa, Moses Egesa, Emmanuella Driciru, Jan Pieter R Koopman, Ronald Kiyemba, Richard E Sanya, Jacent Nassuuna, Agnes Ssali, Geofrey Kimbugwe, Anne Wajja, Govert J van Dam, Paul L A M Corstjens, Stephen Cose, Janet Seeley, Dorcas Kamuya, Emily L Webb, Maria Yazdanbakhsh, Pontiano Kaleebu, Afzal A Siddiqui, Narcis Kabatereine, Edridah Tukahebwa, Meta Roestenberg, Alison M Elliott

**Affiliations:** MRC/UVRI & LSHTM Uganda Research Unit, Entebbe, Uganda; London School of Hygiene & Tropical Medicine, London, UK; MRC/UVRI & LSHTM Uganda Research Unit, Entebbe, Uganda; London School of Hygiene & Tropical Medicine, London, UK; MRC/UVRI & LSHTM Uganda Research Unit, Entebbe, Uganda; Leiden University Medical Center, Leiden, The Netherlands; MRC/UVRI & LSHTM Uganda Research Unit, Entebbe, Uganda; MRC/UVRI & LSHTM Uganda Research Unit, Entebbe, Uganda; African Population and Health Research Center, Nairobi, Kenya; MRC/UVRI & LSHTM Uganda Research Unit, Entebbe, Uganda; MRC/UVRI & LSHTM Uganda Research Unit, Entebbe, Uganda; London School of Hygiene & Tropical Medicine, London, UK; MRC/UVRI & LSHTM Uganda Research Unit, Entebbe, Uganda; MRC/UVRI & LSHTM Uganda Research Unit, Entebbe, Uganda; Leiden University Medical Center, Leiden, The Netherlands; Leiden University Medical Center, Leiden, The Netherlands; MRC/UVRI & LSHTM Uganda Research Unit, Entebbe, Uganda; London School of Hygiene & Tropical Medicine, London, UK; MRC/UVRI & LSHTM Uganda Research Unit, Entebbe, Uganda; London School of Hygiene & Tropical Medicine, London, UK; Kenya Medical Research Institute (KEMRI), Kilifi, Kenya; London School of Hygiene & Tropical Medicine, London, UK; Leiden University Medical Center, Leiden, The Netherlands; MRC/UVRI & LSHTM Uganda Research Unit, Entebbe, Uganda; London School of Hygiene & Tropical Medicine, London, UK; Texas Tech University Health Sciences Center, Lubbock, TX, USA; Ministry of Health of Uganda, Kampala, Uganda; Ministry of Health of Uganda, Kampala, Uganda; Leiden University Medical Center, Leiden, The Netherlands; MRC/UVRI & LSHTM Uganda Research Unit, Entebbe, Uganda; London School of Hygiene & Tropical Medicine, London, UK

**Keywords:** human-controlled Schistosoma masoni

## Abstract

Control of schistosomiasis depends on a single drug, praziquantel, with variable cure rates, high reinfection rates, and risk of drug resistance. A vaccine could transform schistosomiasis control. Preclinical data show that vaccine development is possible, but conventional vaccine efficacy trials require high incidence, long-term follow-up, and large sample size. Controlled human infection studies (CHI) can provide early efficacy data, allowing the selection of optimal candidates for further trials. A *Schistosoma* CHI has been established in the Netherlands but responses to infection and vaccines differ in target populations in endemic countries. We aim to develop a CHI for *Schistosoma mansoni* in Uganda to test candidate vaccines in an endemic setting. This is an open-label, dose-escalation trial in two populations: minimal, or intense, prior *Schistosoma* exposure. In each population, participants will be enrolled in sequential dose-escalating groups. Initially, three volunteers will be exposed to 10 cercariae. If all show infection, seven more will be exposed to the same dose. If not, three volunteers in subsequent groups will be exposed to higher doses (20 or 30 cercariae) following the same algorithm, until all 10 volunteers receiving a particular dose become infected, at which point the study will be stopped for that population. Volunteers will be followed weekly after infection until CAA positivity or to 12 weeks. Once positive, they will be treated with praziquantel and followed for one year. The trial registry number is ISRCTN14033813 and all approvals have been obtained. The trial will be subjected to monitoring, inspection, and/or audits.

## Introduction

Schistosomiasis is a leading neglected tropical disease in terms of disability-adjusted life years (DALYs) [1]. Globally, about 252 million people are infected, over 90% of whom live in Africa, Latin America, and South-East Asia [2]. In the tropical regions of these low- and middle-income countries, schistosomiasis is endemic because of the presence of freshwater snails combined with poor sanitation, but this disease can also spread to sub-tropical areas such as the island of Corsica in Europe [3]. Tourists to tropical areas can be affected, sometimes with severe sequelae such as transverse myelitis [4].

The life cycle of *Schistosoma* species that infect humans includes asexual reproduction within the snail intermediate host, with parasites released as infective and swimming larvae, known as cercariae. The cercariae enter the skin, lose their tail (transforming into schistosomula) and migrate through the bloodstream and lungs reaching the portal or peri-vesical vasculature. Here, schistosomula mature into adult male and female worms which pair, mate, and release eggs in the mesenteric or bladder venules. Eggs released into the environment through the digestive or urinary tract hatch and infected miracidia may infect susceptible snails, continuing the cycle [5].

The human pathology from schistosomiasis mainly occurs in high burden infections when the eggs are unable to exit from the human host, and lodge in tissues causing inflammatory responses, granulomas, and subsequent fibrosis. These changes are responsible for long-term pathologies such as portal hypertension and hepatosplenomegaly in the case of *Schistosoma mansoni*, and haematuria, urinary tract infections, hydronephrosis, and kidney failure in *S. haematobium* infection. Inflammation of the female reproductive tract may cause genital lesions that are risk factors for acquiring sexually transmitted diseases such as HIV [6].

Currently, treatment and control of schistosomiasis is dependent on praziquantel, usually given as a 40 mg/kg single dose. There is substantial variability in cure rates with estimates ranging from 40 to 90% [7, 8]. This is mostly influenced by the timing after the initial infection, the pre-treatment parasite load and the intensity of exposure to reinfection [9, 10]. Repeated praziquantel dosing may thus be required to achieve a full cure. Mass drug administration, used in endemic settings, does not prevent reinfection and in exposed populations, the disease prevalence returns to its original level within 6–8 months [11]. There are also concerns about the possible emergence of drug resistance following repeated intervention with a single drug [12].

The development of a vaccine against schistosomiasis would be a valuable tool in the control of this important parasitic disease, with vaccine-induced immunity providing protection against repeated reinfection [13, 14]. Vaccination studies in mice and non-human primates with radiation-attenuated cercariae have provided the strongest proof-of-concept that vaccination against schistosomiasis is feasible [15, 16]. Stage-specific parasite antigens have been identified as vaccine candidates, aiming to prevent infection or reduce worm burden and egg excretion. The pathway for WHO endorsement of these candidate vaccines would be to generate at least a 40% reduction in worm burdens [17]. To date, four vaccine candidates (Sh28GST in Alum formulation [18], Sm-TSP-2 combined with Alhydrogel and/or GLA [19], Sm14 [17] and Sm-p80 with GLA-SE adjuvant [20]) are in the clinical stage of development. Normally, these vaccine candidates would have to go through trial testing requiring populations with a high incidence of *Schistosoma* infection, long duration of follow-up and large sample sizes for sufficient power to demonstrate efficacy. However, controlled human infection studies have the capacity to demonstrate efficacy quickly, in a small number of participants, as seen in well-established models for vaccine candidates for malaria, dengue, and influenza [21]. Funding for schistosome vaccine development is limited, therefore, “down selection” of the most promising candidates would be cost-effective ahead of large trials.

In the current study, we aim to contribute to the development and implementation of a controlled human infection model for schistosomiasis (CHI-S) that can be used to test innovative and early proof-of-concept candidate schistosomiasis vaccines and study *Schistosoma* immune responses. The first-in-human proof-of-concept study has been successfully conducted at Leiden University Medical Center [22] but we predict that responses both to *Schistosoma* infection and to vaccines will differ in the primary target populations in endemic settings, as discussed below [23]. We will establish the model in an endemic country, Uganda that would be a primary beneficiary of *Schistosoma* vaccines. Estimates show that in Uganda around a quarter [24] of the population is infected and half [25] is at risk of infection. To avoid the risk of egg-induced pathology, particularly neuroschistosomiasis, the strategy of developing single-sex (in this case male-only) CHI-S models for schistosomiasis has been adopted [26].

In the Leiden study, to prepare inoculum, laboratory snails were infected, each with a single miracidium, resulting in many single-sex cercariae; the male sex was determined by real-time multiplex PCR for the schistosome-specific ITS2 gene and the female-specific W1 gene [26]. In the dose-finding studies, *S. mansoni*-naïve Dutch volunteers were infected with 10, 20, or 30 male cercariae [26, 27]. Consistent infection, quantifiable using a highly sensitive serum assay for circulating anodic antigen (CAA) excreted from the gut of adult worms [28], was detectable in 80% of participants exposed to 20 cercariae. The infection was safe and tolerable. Adverse events occurred including Katayama syndrome, which responded well to paracetamol, NSAIDS, and cetirizine treatment [26]. In the immunological analysis, the production of high levels of IgG1 antibody to many different schistosome-specific protein and glycan antigens and both IFNɣ and Th2-cytokine were observed. Of note, among exposed individuals in endemic areas, acute schistosomiasis syndrome does not occur [5].

### Transfer of CHI-S to endemic settings

Transfer of the CHI-S to an endemic setting such as Uganda is needed to shed light on the differences in immune response between unexposed European and previously exposed Ugandans. Prior exposure to schistosomiasis influences schistosome-specific immune responses [29, 30]. In addition, a myriad of other infectious and environmental exposures differs between temperate and tropical settings and impact the activation profile of the innate and adaptive immune response [31], so Ugandan research participants are likely to respond differently to European research participants, even if they are also schistosome-unexposed. Moreover, the response to, and efficacy of, schistosome candidate vaccines is also expected to differ between settings. For example, efficacy may be masked by partial protective immunity induced by prior schistosome exposure or modulated by differences in pre-immunisation immunological activation profile. The critical test of preliminary efficacy will be in the target, schistosomiasis-endemic communities.

Because of the immunological differences between populations, the dose of cercariae needed to achieve infection in the CHI-S is expected to differ between Leiden-Netherlands and Entebbe-Uganda, and between communities in Entebbe with minimal, or intense, prior schistosome exposure. The CHI-S studies proposed here will provide opportunities to explore both non-specific and antigen-specific correlates of immunity to infection. In this trial, we will focus on controlled human infection with *S. mansoni*, which is responsible for the majority of morbidity due to schistosomiasis worldwide, and in Uganda.

## Methods

### Protocol objectives

#### Primary objective

To investigate the safety, tolerability, and infectivity of male *S. mansoni* cercariae in healthy adult Ugandan volunteers: (1) with minimal prior exposure to *S. mansoni*, and (2) with intense prior exposure (living on Lakeshore with antibody data showing past exposure) to *S. mansoni*.

#### Exploratory objectives

To investigate the kinetics of controlled infection with male *S. mansoni* cercariae in healthy adult Ugandan volunteers: (1) with minimal prior exposure to *S. mansoni*, and (2) adults with intense prior exposure to *S. mansoni*.

To investigate immunological, metabolic, and microbiome changes after infection with *S. mansoni* male cercariae.

To investigate volunteer and wider community understandings of CHI in the context of CHI-S:

(1) To assess volunteers’ and wider community responses to CHI-S in Uganda.
(2) To compare experience on the community response to CHI for other pathogens and in other countries – e.g. with experience in Kenya on CHI studies for malaria and Shigella.

### Experimental design

**Figure 1.**
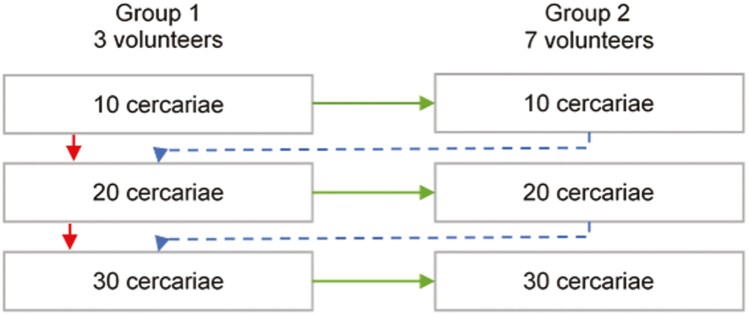
Step-wise dose escalation diagram. At each dose of cercariae, three volunteers will be infected in group 1. If safety profiles are acceptable at 8 weeks from infection, and all three are infected, a second group of seven volunteers will be infected at the same dose (group 2; green arrows). If safety is acceptable but not all three are infected dose will be escalated (red arrows), If safety profiles remain acceptable, the dose will be escalated until at least eight of ten volunteers are infected at a given dose.

This will be an open-label, dose escalation intervention trial. Dose escalation will be done separately for the two populations (minimal prior exposure and intense prior exposure). Trial volunteers will be exposed to predefined doses of cercariae in groups of three and subsequently seven volunteers. Dose escalation will be performed according to the schedule depicted in Fig. 1. If all three volunteers who first receive a specific dose show evidence of infection (based on detectable circulating anodic antigen (CAA) in serum), seven additional volunteers will be exposed to the same dose. If not all ten volunteers in a dose group become infected, the next group of three volunteers will be exposed to a higher dose. If all ten volunteers receiving a dose then become infected, there will be no further dose escalation and the study will be stopped in that population. All volunteers included in the trial will be followed as outpatients for one year. They will be followed on a weekly basis after infection until CAA positivity is confirmed (expected between approximately 6 and 8 weeks). Once CAA positive (or, at the latest, at 12 weeks after exposure to infection) volunteers will be treated with praziquantel 40 mg/kg. Volunteers will also be seen at weeks 14, 16, 18, 20, 22, and 24 and one year after infection. A second treatment with praziquantel at 60 mg/kg will take place 4 weeks after the first dose if volunteers remain positive.

### Volunteer treatment

Volunteers will be exposed to a predefined dose of male cercariae once according to the schedule depicted in Fig. 1, by applying male cercariae to the intact skin of the forearm for 30 min. Cercariae will be applied to the skin in 0.5 ml Rwenzori or Bar-le-duc water. After this exposure, treatment with triamcinolone cream 0.5% or a similar product per local clinical practice [32] will be provided in case of severe itching at the site of entry of larvae (swimmer’s itch). In the event of Katayama syndrome (symptoms of acute schistosomiasis infection), the volunteer will receive appropriate treatment such as paracetamol, non-steroidal anti-inflammatory agents or inhaled β2 agonists (Ventolin), inhaled corticosteroids or oral prednisolone in case of severe symptoms lasting more than 48 h.

A trial physician may decide to abrogate the study for individual research participants in symptomatic cases, and provide treatment with artemether/lumefantrine in a standard 6-dose treatment regimen over 3 days, together with praziquantel 40 mg/kg, for individual volunteers in weeks 0–10 of the study; or praziquantel 60 mg/kg for those in weeks 10–12 of the study.

### Justification of the route of administration, dosage, and dosage modification

In the CHI-S model, the male cercariae penetrate the skin of human volunteers. This is similar to the natural route of infection and is safe as complications at the site of entry other than swimmer’s itch (an urticarial rash) have not been observed or documented. We expect that about 50% of cercariae will develop into mature worms after penetration of the skin as evidenced from studies in non-human primates [33] and rodents [34]. We estimate that about five mature male worms are needed to potentially detect circulating CAA because not all cercariae may penetrate the skin. Based on these considerations, and safety data from Leiden [27], we chose to take a few cercariae as the minimal dose for controlled human infection that may possibly be detectable in a number of volunteers.

### Cercariae preparation

A technician will prepare doses of cercariae by manual counting which will then be checked by a second technician. Cercariae will be prepared in the A1 well of a 24-well plate and administered. After controlled human schistosomiasis infection, the number of tails, heads, and intact cercariae will be counted under the microscope and numbers noted in the clinical trial database. All disposable equipment used will be destroyed according to biosafety level 2 regulations.

### Community engagement

Before initiating this work, we held a Stakeholders’ meeting in November 2017 [35]. Discussion included the identification of appropriate communities. Based on colleagues’ experiences in Kenya with the implementation of the malaria model, it was proposed to include a university community (but not students still dependent upon their parents), in the expectation that volunteers’ level of education would make it easier for them to understand the proposed work [35]. Ultimately, however, the target community of most interest will be residents of settings with intense schistosome exposure.

In the roadmap for establishing the CHI-S for Uganda, which arose from the Stakeholders’ meeting, a key step identified was to develop processes which would ensure that volunteers understood the CHI-S thoroughly. It was also important to ensure that it would be possible to support volunteers to comply with the trial requirements, particularly avoidance of exposure to natural infection. Educational materials have been developed for this together with volunteer information sheets in English and Luganda, and tests of comprehension. These have been refined in discussion with target community members [36].

### Study population

We will conduct this dose-finding and safety study among adults from two Ugandan communities, one with minimal and one with intense prior schistosome exposure. Both types of community are targets for a future vaccine, the former to prevent infection on initial exposure, the latter to reduce reinfection following a treat-and-immunise strategy. We hypothesise differences between the communities in the CHI-S dose required (higher in intense-exposure communities due to partial immunity) and in challenges to participation (prior knowledge and attitudes to schistosomiasis, vaccines, and trials; ability to comply with study requirements; appropriate levels of compensation). Healthy research participants, male or female, aged 18–45 years old will be included in the study.

**Figure 2.**
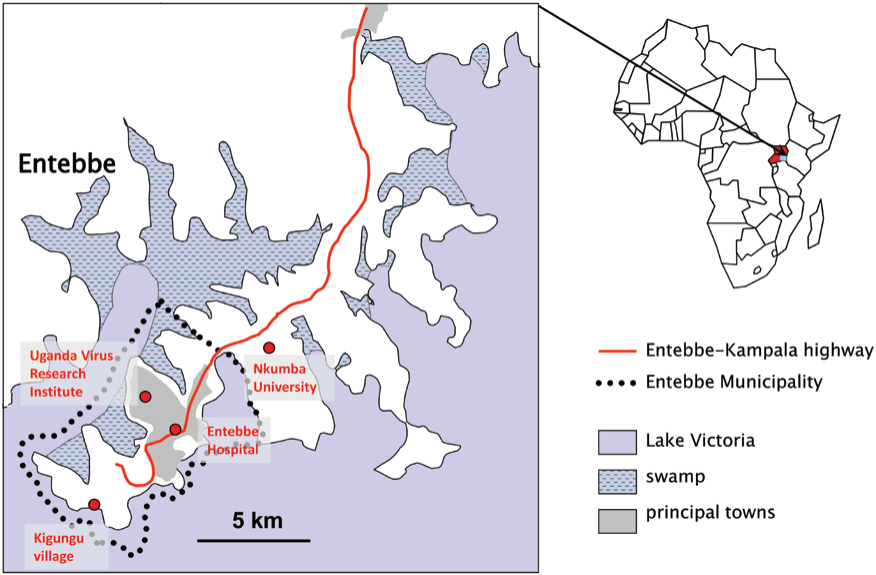
Trial setting illustrating the location of the clinical research facility that will house the trial work and the two communities, prior minimal schistosome exposure (Nkumba University) or intense schistosome exposure (Kigungu—Lakeshore fishing village) from which the volunteers will be obtained.

Trial work will be based at the MRC/UVRI and LSHTM Uganda Research Unit (MUL) at Uganda Virus Research Institute (UVRI). These facilities offer strong capacity for trials, and laboratories equipped for outcome assessments (Fig. 2). We propose to enrol volunteers from two categories of community—an institution of higher education (within easy reach of the Institute, Nkumba University); and a Lakeshore fishing village, (Kigungu). Engagement is ongoing with members of these communities. The University attracts students and staff from across Uganda and is expected to contribute volunteers with minimal prior schistosome exposure and tertiary-level education, who are able to understand the work well. Based on our earlier Stakeholders’ meeting, we shall not enrol students still dependent upon their parents [35], but mature students, academic and administrative staff will be eligible. Fishing communities on the Entebbe peninsula are expected to contribute volunteers with intense prior exposure, but with access to piped municipal water supplies and adequate sanitation, able to participate safely, and avoid natural superinfection during the studies.

Planned inclusion and exclusion criteria are listed in Table 1.

**Table 1.** Inclusion and exclusion criteria

Inclusion	Exclusion
• Age ≥18 years and ≤45 years	• Current *Schistosoma* infection (CAA assay ≥ 0.8 pg/ml)
• Available and able to communicate effectively	• Malaria or intestinal helminth infections
• Able to understand study procedures	• If female, positive urine pregnancy test at screening
• Planning to remain in the study areas for 16 + weeks and be reachable by mobile phone	• Known hypersensitivity to or contra-indications for the use of praziquantel, artesunate, or lumefantrine
• Willing to avoid contact with waterbodies for 12–16 weeks and until the controlled infection is cured	• Chronic use of any drug known to interact with praziquantel, artesunate, or lumefantrine metabolism
• Willing to refrain from blood donation throughout of the study period	• Participation in another research involving receipt of an investigational product in the 30 days preceding enrolment
• If the female, is willing to use adequate contraception and not to breastfeed for the duration of study	• History, or evidence at screening, of clinically significant symptoms, physical signs or abnormal laboratory values suggestive of systemic conditions, such as cardiovascular, pulmonary, renal, hepatic, neurological, dermatological, endocrine, malignant, haematological, infectious, immune-deficient, psychiatric, and other disorders, which could compromise the health of the volunteer during the study
• Willing to sign written informed consent	• An employee or student of the Uganda Virus Research Institute or its partners

### Management of conditions identified during screening and study period

Volunteers found to have health conditions that need treatment will be informed, counselled and provided with treatment, or referred for appropriate healthcare services. After treatment, participants may be reconsidered for inclusion after the cure. For the intense-exposure community, volunteers who are CAA positive will be treated until CAA negative (CAA level <0.8 pg/ml) prior to enrolment. There will be a period of waiting and retesting thereafter to ensure that there is no recovery of sick worms or maturation of young worms.

If a woman does become pregnant during the trial, she will continue to undergo blood tests for CAA detection on a weekly basis and will be treated with praziquantel 40 mg/kg as soon as the test becomes positive. If CAA does not become positive, presumptive treatment with praziquantel will be delayed until the end of the first trimester of pregnancy. Adverse effects of praziquantel on the mother or fetus are not expected; treatment of schistosomiasis during pregnancy is considered acceptable by WHO for mass drug administration campaigns [37]. Artemisinins will be avoided in the first trimester of pregnancy as there are only limited data on their safety in the first trimester [38]. The volunteer will be followed up to delivery to ascertain her health and that of her baby.

### Sample size calculation

For future vaccine proof-of-concept efficacy studies, small groups of roughly 10 research participants are generally preferred, allowing for the detection of roughly ~50% protective vaccines with a power of 80% (alpha < 0.05, two-tailed). A full 100% infection rate in the infectivity control group is required to achieve this power. With a 90% infection rate in the control group, there is 80% power to detect 62% protection in the vaccine group. Therefore, we aim in this study to achieve a 100% infection rate in 10 research participants.

### Strain, and justification of, the strain of *S. mansoni* to be used

Male cercariae from the laboratory strain of *S. mansoni* which has been maintained in Leiden for over 50 years will be used. This strain was originally isolated from Puerto Rican samples [27]. This is considered to be safer for volunteers than the use of a local strain generated from fresh Ugandan isolates. The Puerto Rican laboratory strain is inbred, making the characteristics of any particular clone, including virulence and susceptibility to praziquantel, predictable. A new local (Uganda) isolate would involve a population of outbred schistosomes, and mass drug administration has applied some pressure on praziquantel usage in Ugandan communities [12]; virulence and praziquantel susceptibility of clones generated for human infection would be less predictable until the new laboratory strain was well-established.

### Summary of known and potential risks and benefits

There is no direct benefit of participation in this study to the volunteer. The risks associated with participation in this study are those related to infection with male *S. mansoni.* An urticarial rash (Swimmer’s itch) at the point of entry of the cercariae and Katayama syndrome with symptoms such as fever, fatigue, myalgia, malaise, non-productive cough, and eosinophilia may occur [39]. Only a mild form of Swimmer’s itch is expected to occur since a small number of cercariae will be used. Also, the chances of developing Katayama fever are minimal in endemic settings like Uganda [5]. Pathologies caused by egg production and deposition, such as abdominal symptoms and hepatosplenomegaly, are not expected to occur since single-sex (male-only) cercariae will be used. All the above conditions are treatable with triamcinolone cream 0.5% or similar products, paracetamol, NSAIDS, (oral) corticosteroids, artemisinins, and praziquantel as detailed above.

### Trial procedures

#### Recruitment of volunteers

At the University, advertisements will be placed in prominent places on the campus and other public places. Short seminar presentations will be held and flyers provided indicating a telephone number to call and an e-mail address for contact to request further information. At the fishing community, meetings will be held with community leaders and village members to explain the work.

Subsequently, the study team will hold further meetings aimed at providing potential volunteers with more detailed information (the educational material and the information sheet about the study). Volunteers interested in joining the study will be requested to consult at least a family member and/or close friend, who will be a documented contact person during the study. Following the successful completion of these discussions and receipt of informed consent from the volunteer, an appointment for screening into the study will be scheduled at least 72 h after consent.

#### Screening visit

The study team will engage the volunteer in the review of the study information sheet, sign the informed consent, administer the test of study comprehension, discuss the responses and check the inclusion–exclusion criteria. Additionally, the study team will respond to any queries the volunteer might have. Subsequently, a physical examination will be performed, with blood, urine, and stool samples taken as shown in Table 2.

**Table 2.** Study procedures to be completed at indicated study visit week

CHI dose escalation and safety							
Visit (V)	Screening	V1	V2–13	V14	V15–17	V18–20	V21
Week	–30	0	1–11	12	14, 16, 18	20, 22, 24	52
Deviation	–60/+15 days^*^		±3 days	±3 days	±3 days	±1 week	±4 weeks
Information and consent	Information, sign consent	Discuss; review/affirm consent					
Clinical history and examination, AE collection	X	(x)	(x)	(x)	(x)	(x)	(x)
Height	X						
Weight	X			x			
Vital signs (temperature and blood pressure)	X	x	X	x	X	x	x
Exposure to cercariae		x					
Praziquantel				x	x^3^		
CAA (serum)^1^	x^4^	x	X	x	X	x	x
Safety tests							
Urine dipstick (general health check)	X						
CBC/eosinophils	X		weeks 6, 8, 10	x	weeks 14, 16	x	x
Liver function tests (LFTs): bilirubin, AlkP, γGT, and AST, ALT	X			x			
Renal function tests (RFTs): creatinine, urea, sodium, and potassium	X			x			
Glucose	X			x			
Pregnancy test for women	X	x	week 8	x	week 16		
Co-infections							
HIV	X						
HBV	X						
HCV	X						
Malaria RDT	X						
Malaria PCR	X						
Stool PCR (multiplex helminths)	X						
Stool storage for microbiome	X		X	x	X	x	x
Store serum	X	x	X	x	X	x	x
Serology for prior exposures^2^	X						
Immunological investigations and storage of cells	X	x	weeks 4, 6, 8	x	X	x	x
Metabolomics^1^	X	x	X	x	X	x	x

(x) targeted examination as indicated by history.

^*^Safety tests and co-infections repeated if tests conducted >30 days before exposure to infection.

1: weekly.

2: serology for Schistosomiasis (worm and egg ELISA) and other prior infections, e.g. malaria.

3: repeated if CAA remains positive.

4: repeated, if initially positive, until consecutive tests are negative.

### Infection with male *S. mansoni* cercariae

At week zero (day of exposure to male *S. mansoni* cercariae), volunteers will visit the clinical trial centre in the morning where a final check of the inclusion–exclusion criteria and other required week zero procedures shown in Table 2 will be done. A blood sample will be drawn for repeat CAA and immunological assays. On the same day, volunteer exposure to male *S. mansoni* cercariae will be performed at the trial clinic by a trained study staff member. Male cercariae will be allowed to penetrate the skin of volunteers by applying 0.5 ml of Rwenzori or Bar-le-duc water containing the specified number of cercariae on the skin for 30 min. A second study staff member will cross-check the procedures to ensure accuracy and completion of the study procedures, after which the volunteer will be observed for at least 30 min.

#### Follow-up after infection

Following inoculation with male *S. mansoni* cercariae, volunteers will visit the trial centre weekly for 12 weeks and thereafter biweekly until week 24. All visits will be completed as shown in Table 2 until the final study visit at week 52 following infection. At each clinic visit, the procedures shown in Table 2 will be completed. A clinical trial physician will be available at the trial clinic and by mobile phone 24 h a day for volunteers to report any adverse events needing attention to enable the detection and treatment of symptoms of acute schistosomiasis infection. Treatment with praziquantel may be provided but also at the physician’s discretion, diagnostics including serum CAA tests can be performed.

#### Safety laboratory evaluation

 Laboratory safety tests are detailed in Table 2. Key safety analysis will include complete blood count (including an automated differential count of white blood cells), creatinine, potassium, bilirubin, and liver enzymes. Biological safety parameters will be measured on plasma or serum samples at the Clinical Diagnostic Laboratory (CDLS) of the MRC/UVRI and LSHTM Uganda Research Unit in Entebbe Uganda. On the other hand, an assessment of successful infection will be performed by serum CAA measurements at the immunology laboratories of the Immunomodulation and Vaccines Programme of MUL. Furthermore, *S. mansoni* antigen detection tests will be performed prospectively on all samples (on the same day for the baseline sample, but generally within one week of obtaining the sample). The objectives are first, to exclude active infection prior to the start of the CHI study and second, to enable any infection to be abrogated as soon as it is detected.

#### Source data

All data collected by the investigator will be reported in electronic case report forms. These forms, together with the investigator’s notes are considered source data. In case of adverse events or reactions resulting in a medical consultation or hospitalization, a medical file will be made. In this case, the medical file will be considered as the source data.

#### Adverse events

Signs and symptoms will be recorded at all visits and whenever a trial volunteer reports signs or symptoms to the trial physician between visits. A clinician to handle these will be available as indicated above. The following signs and symptoms will be solicited at all visits: itching, fever (by examination), rash, urticaria, headache, fatigue, malaise, coughing, myalgia, arthralgia, night sweats, back pain, anorexia, nausea, vomiting, abdominal pain, and diarrhoea.

### Key study endpoints

The primary endpoints will be: (a) frequency and magnitude of adverse events after controlled human *S. mansoni* infection with male cercariae, (b) the number of male cercariae at which 100% of volunteers show detectable *S. mansoni* circulating anodic antigen. Other study parameters will include (c) time to positive serum CAA test, (d) a comparison of the height of the peak serum CAA concentration in different dose groups, (e) humoral responses directed against *S. mansoni* antigens, (f) cellular responses directed against *S. mansoni* antigens, and (g) changes in microbiome after controlled human *S. mansoni* infection with male *S. mansoni* cercariae.

### Volunteer and wider community understandings of CHI in the context of CHI-S

The MRC/UVRI and LSHTM research team as well as colleagues in the Ministry of Health’s Vector Control Division, have worked intensively with fishing communities [40–42]. They have explored attitudes to schistosome vaccine studies and trials, and these were found to be positive [41, 42]. In this study, the social science team will work with the community engagement team to assess volunteers’ and the wider community’s understanding of CHI-S in Uganda. Selected volunteers will be invited to participate in three semi-structured interviews, one after consenting to take part, one at week 20 and the last one at the time of exit from the trial. The interviews will investigate perceptions and knowledge of trial procedures, and expectations, and experiences of the trial. Additionally, 10 single interviews will be conducted with non-participants once the trial is completed to gather their views. The interviews will be conducted by experienced social scientists, recorded, transcribed, and translated. The social science team will compare findings from the CHI-S study with data collected by colleagues in Kenya, where work is being conducted on other models, including CHI for malaria and Shigella. We will analyse similarities and differences experienced and lived by volunteers in the different contexts to draw lessons for future vaccine trial implementation,

### Exploratory immunology and metabolomics

The overall objective of this exploratory work is to find immunological or metabolic markers that associate with male *S. mansoni* infection. The sample collection schedule is shown in Table 2. Humoral assessment will include antibody assays by immunofluorescence and/or ELISAs and/or antibody arrays for specific *S. mansoni* proteins or glycans. Cellular assessment of parasite-specific T-cell responses will be conducted by multi-parameter flow cytometry, CyToF and ELISPOT assays with or without using *S. mansoni*-specific *in vitro* stimulation. Similarly, innate immune responses associated with susceptibility to schistosome infection will be assessed.

RNA will be extracted from PAXgene samples collected before and after CHI-S, for transcriptomic analysis. Identifying worm-specific immune responses induced by the challenge will be integral to understanding protective immunity. Signatures will be compared with those obtained in related studies in the Netherlands and elsewhere. Assays will be conducted in Uganda when appropriate facilities are available. More complex immunological work (e.g. using mass cytometry time-of-flight; CyToF) will, if necessary, be conducted in the Netherlands at Leiden University Medical Centre, and RNAseq will, if necessary, be conducted in the USA at Texas Tech University Health Sciences Center. Ugandan scientists/trainees will travel to these centres to learn the techniques. The metabolic profile of serum and urine samples will be evaluated at time points before, during and after infection if funds allow. An analysis of faecal samples to investigate the gut microbiome will be performed at 5-time points during the trial if funds allow.

### Analysis

#### Primary study statistical analysis

All volunteers exposed to male *S. mansoni* cercariae will be examined in the assigned group in an intention-to-treat (ITT) analysis for adverse events. Per protocol, analysis will also be performed considering volunteers completing the follow-up until 12 weeks after infection. Adverse events will be summarised per volunteer and group in an ITT analysis to describe the safety and tolerability of controlled infection with male *S. mansoni* cercariae. The adverse events will be reported by severity status; mild, moderate or severe events. Where appropriate, Fisher’s exact test will be used to compare between groups. A descriptive table will be used to summarise volunteers’ data with patent infection (a positive CAA test (≥1.0 pg/ml), between 0 and 12 weeks following infection with *S. mansoni* male cercariae or those withdrawn because of infection). Time to positive serum CAA test will be examined using a Kaplan–Meier plot and log-rank (Mantel–Cox) test and compared between different dose groups. In the immunological analysis, parametric or non-parametric tests will be used as appropriate to compare means/medians between the dosage groups.

Qualitative data will be analysed using a thematic framework approach. The social science team members will agree on codes from the data, come up with a coding frame and code the data, using constant comparison to ensure inter-coder consistency. Codes will be drawn both deductively and inductively from the trial aims and those arising from the data. Coded data will be grouped into broad themes. Thematic summaries will be developed and shared with the wider research team for ongoing discussions throughout the study and for final write-up.

## Discussion

The aim of this protocol is to assess the safety, tolerability, and infectivity of male *S. mansoni* cercariae in a controlled human infection model for schistosomiasis (CHI-S) among healthy adults with minimal and those with intense prior exposure to *S. mansoni* who are residents of an endemic country.

The justification for this model for schistosomiasis lies in its potential to accelerate the vaccine development pipeline by enabling early selection of effective vaccine candidates [27] as seen in the contribution of malaria and typhoid CHI models to the development of new malaria and typhoid vaccines [21]. Once established, the CHI model is expected to be cost-effective, compared to classical Phase III clinical trials, in rapid testing of vaccine efficacy, and exploration of correlates of protection, and requires a small sample size. Unlike natural infections, CHI enables the study of the natural history of infection/host–pathogen interactions, evolution of immune responses and assessment of vaccine efficacy using well-defined timing of the exposure such that all the signs and symptoms of disease, and evolution of responses, can be well described and managed. Hence, CHI provides a unique opportunity to conduct in-depth mechanistic studies into infection-driven protection in the human host. This is particularly important for schistosomiasis, where animal models do not accurately depict the pathophysiology of infections in humans to inform the vaccine development [21].

This programme is the first to seek to establish a CHI-S model in Africa. As a result, careful consultations with stakeholders and communities have been essential [35, 36]. We have also engaged the Uganda Ministry of Health and national regulators to build confidence and appreciation of the model and to obtain their support. Discussions have been held to help identify the most appropriate communities. Colleagues from other African countries where CHI for various pathogens have been established, including their regulators and ethicists, have provided important guidance [35].

Schistosomiasis CHI has several characteristics distinct from other CHI models. The pathogen-specific adverse events such as swimmer’s itch, Katayama fever, and acute toxemic neuroschistosomiasis among others may occur—some of which were seen in the Dutch trials in Leiden [27] —but these may be fewer in Uganda due to endemic *S. mansoni* infections and distinct population immune profile [23]. For *S. mansoni* there is no commercially available infection product to be used in trials, leading to lengthy processes for setting up and seeking approvals, particularly relating to the unique environmental risks, that could be associated with importing non-endemic schistosome and snail species. If in humans, co-infection with the Puerto Rican schistosome strain and Ugandan strains occurs, this may lead to hybridisation with unknown consequences for infectiousness and pathology. Given the Dutch experience of infection in humans and known sensitivity to praziquantel, it is unlikely that consequences would be severe. However, to minimise the risk of co-infections, we shall carefully select and educate possible volunteers, and early abrogation of infection will be used to minimise this risk. On the other hand, importation of the non-native *Biomphalaria glabrata* snail species used in Leiden will require sound containment processes in the laboratory, since escape into the environment could have important consequences for snail and schistosome ecology [43].

Many stakeholder and community engagement activities have already been completed to ensure stakeholders and volunteers understand the CHI-S thoroughly, to achieve high trial compliance, particularly avoidance of exposure to natural infection [35, 36], as well as to enhance the public understanding of science conducted in these communities. Educational materials together with volunteer information sheets have been developed in English and the local language, and tests of comprehension conducted. The test of comprehension to be administered will have ten questions (three on schistosomiasis and vaccines) and (seven on controlled human infection studies). The questions on the controlled human infection studies were designed to capture salient features of controlled human infection studies including expectations from the volunteers during the study, potential symptoms, and benefits. These have also been widely shared and discussed with the targeted community members [36]. Engagement activities will be sustained throughout the trial to address any emerging concerns and responses from the stakeholders and communities.

This protocol is not without limitations including difficulty to distinguish natural from controlled infection if a volunteer is exposed to water bodies despite caution to avoid this. We will conduct weekly antigen and stool sample tests to detect and immediately treat any infections. If only antigen is detected, it will not be possible to distinguish between natural and controlled infection. In exceptional circumstances, if eggs are detected in stool, it may be possible to use these to obtain parasite DNA and explore whether the infection is purely natural or mixed, by genetic means.

## Data Availability

The MRC/UVRI and LSHTM Uganda Research Unit operates an open data access and the Unit’s data sharing policy is accessible at https://web.archive.org/web/20201201165922/http://www.mrcuganda.org/sites/default/files/publications/MRC_UVRI_Data_sharing_policy_December2015.pdf. The policy summarises the conditions under which data collected by the Unit can be made available to other bona fide researchers, the way in which such researchers can apply to have access to the data and how data will be made available if an application for data sharing is approved. The corresponding and other co-author email addresses will be provided for contact at any time, and for further clarifications and/or support to access the data.

## Abbreviations:

CAA: Circulating anodic antigen
CHI: Controlled human infection
DALYs: Disability-adjusted life years
DNA: Deoxyribonucleic acid
ELISA: Enzyme-linked immunoassay
HBV: Hepatitis B virus
HIV: Human immunodeficiency virus
ISRCTN: International standard randomised controlled trial number
ITT: Intention to treat
LSHTM: London school of hygiene & tropical medicine
MRC: Medical research council
MUL: MRC Uganda limited
PCR: Polymerase chain reaction
RDT: Rapid diagnostic test
*S. mansoni*: *Schistosoma mansoni*
USA: United States of America
UVRI: Uganda Virus Research Institute
WHO: World Health Organisation

## Participants and public involvement

Public input was obtained during a public engagement meeting. Members of the public reviewed study information i.e. the information sheets and consent data.

## Clinical trial registration

The trial is registered under number: ISRCTN14033813.
